# RBM5 Acts as a Tumor Suppressor in Breast Cancer Through Binding to G-quadruplexes in the BAP1 Gene Promoter to Activate Its Expression

**DOI:** 10.3390/molecules31142492

**Published:** 2026-07-16

**Authors:** Yingzhou Li, Wenmeng Wang, Guangyue Li, Hai Wang, Suixin Cong, Cuicui Yang, Biao Ma, Dangdang Li, Guangchao Sui

**Affiliations:** 1 College of Life Science, Northeast Forestry University, Harbin 150040, China; yingzhouli@nefu.edu.cn (Y.L.); wangwenmeng@nefu.edu.cn (W.W.); wanghai@nefu.edu.cn (H.W.); congsuixin@nefu.edu.cn (S.C.); ccyang@nefu.edu.cn (C.Y.); 2022112834bma@nefu.edu.cn (B.M.); 2 Institute of Biology, Westlake Institute for Advanced Study, Hangzhou 310030, China; liguangyue@westlake.edu.cn

**Keywords:** RBM5, G-quadruplex, breast cancer, BAP1, tumor suppressor

## Abstract

As a member of the RNA-binding motif protein (RBM) family, RBM5 is a characterized tumor suppressor in lung and prostate cancers, with critical roles in alternative splicing of apoptosis- and cell cycle-related genes. However, its direct capacity to regulate gene transcription remains unreported. Here, we identified that RBM5 was significantly downregulated in breast cancer cells and clinical specimens, especially in the basal-like subtype. RBM5 overexpression attenuated breast cancer cell malignancy, while RBM5 knockdown exerted opposite effects. Among genes with promoter G-quadruplex (G4) motifs, RBM5 was positively correlated with multiple tumor suppressors, including BAP1, but uncorrelated with the oncogene MYC. RBM5 bound to G4 motifs in both MYC and BAP1 promoters, but differentially modulated G4 structure stability: it destabilized MYC-G4 while stabilizing BAP1-G4. Mechanistically, either the RRM1 or the RRM2 domain was sufficient for MYC-G4 binding, whereas both domains were required for BAP1-G4 interaction. RBM5 manipulation regulated endogenous BAP1 but not MYC expression. BAP1 overexpression reversed the protumorigenic effects of RBM5 knockdown in cellulo and tumor growth in a xenograft mouse model. Collectively, we reveal that RBM5 acts as a breast cancer tumor suppressor via directly binding the BAP1 promoter G4 to transcriptionally activate BAP1 expression.

## 1. Introduction

Breast cancer has emerged as one of the most prevalent malignant tumors worldwide in recent years and has become the primary threat to women’s health globally. With advancements in medical research, the diagnosis and treatment of breast cancer have significantly improved. Current treatment modalities primarily include surgical resection, radiotherapy, chemotherapy, endocrine therapy, and targeted therapy. As a result, the mortality rate of breast cancer has significantly declined [1]. However, triple-negative breast cancer (TNBC), an aggressive and heterogeneous molecular subtype lacking estrogen, progesterone, and HER2 receptors, continues to exhibit disproportionately high mortality rates without an effective treatment strategy [2]. Clinically, TNBC demonstrates particularly malignant features, including early recurrence, rapid disease progression, shortened overall survival, high metastatic potential, and poor prognosis [2]. These characteristics collectively make TNBC one of the most challenging clinical entities in oncology practice [3]. Current research on prognostic biomarkers and targeted therapeutic strategies for TNBC remains in the preliminary stages, requiring substantial theoretical and mechanistic elucidation. Therefore, dissecting the molecular pathogenesis of TNBC and developing effective treatment approaches have become critically important priorities in global cancer research.

RNA-binding motif proteins (RBMs) constitute a family of proteins containing one or more RNA recognition motif (RRM) domains. As a critical family of RNA-binding proteins, RBMs predominantly function as regulators of cellular apoptosis and cell cycle control [4]. They are involved in multiple processes related to RNA splicing, translation, and stability maintenance, and play key roles in various biological processes such as gene expression regulation and RNA metabolism. Importantly, they have been implicated in the pathogenesis and progression of numerous cancers [4]. Among the RBM family members, RBM5 has been reported to participate in malignant transformation across various cancer types through its RNA-binding activity. Notably, RBM5 demonstrates specific recognition and binding capabilities for guanine (G)-rich RNA sequences [5,6]. Current research positions RBM5 as a potential tumor suppressor gene in multiple malignancies, where it exerts anti-tumor effects by modulating critical cancer cell behaviors, including proliferation, apoptosis, invasion, and metastasis. The mechanisms of RBM5 in cancer mainly include regulating cell apoptosis, affecting cell cycle progression, and modulating tumor-related signaling pathways. Interestingly, RBM4, another member of the RBM family, has been experimentally confirmed to bind G-quadruplex structures in the promoter regions of the KIT and MYC genes [7]. Mechanistic studies reveal that RBM4, also named LARK, promotes the formation and stabilization of G-quadruplex (G4) structures upon binding, consequently enhancing the transcription of downstream target genes [7].

G4s are secondary structures formed by DNA or RNA sequences rich in tandemly repeated guanines. The basic unit of a G4 is a cyclic planar G-tetrad consisting of four guanines stabilized by Hoogsteen-type hydrogen bonds, and three or more stacked G-tetrads comprising a complete G4 structure. Numerous studies have demonstrated the essential roles of G4 structures in regulating various biological processes, such as DNA replication, gene transcription, pre-mRNA splicing, and RNA stability. Consistently, G4 motifs are highly enriched in DNA replication origins, promoters, 5′-UTRs, and splicing sites. Many cancer-related genes, such as BCL2, MYC, HRAS, and hTERT, can be regulated by promoter G4 structures [8,9,10,11]. We have reported G4-mediated expression of YY1, BAP1, ARID1A, and MAZ [12,13,14,15]. Among these studies, promoter G4s can either activate or repress the expression of target genes. Based on the analyses of 76,156 whole-genome sequences, we discovered that promoter G4s are mostly associated with gene activation [16]. Small molecules that can stabilize or disrupt G4 structures have been developed and have shown promising potential as novel cancer therapeutics in pre-clinical studies [17]. Overall, the dynamic G4 structures in the promoters of regulatory genes contribute to dysregulated gene expression in cancer cells and may also serve as effective targets in cancer therapies.

BRCA1-associated protein 1 (BAP1) is a deubiquitinase and participates in diverse biological processes such as cell cycle regulation, differentiation, apoptosis, gluconeogenesis, and DNA damage response. As an important tumor suppressor, BAP1 is strongly implicated in the pathogenesis of multiple cancers [18]. We discovered that the BAP1 promoter harbors G4 structures that positively regulate its gene expression [13]. However, the transcription factors that bind to the G4 structures and mediate BAP1 gene expression have not been elucidated.

Many RBMs have been reported to regulate various RNA-related processes, including pre-mRNA alternative splicing, RNA modifications, RNA stability, and mRNA translation [4]. RBM4 and RBM25 bind G4 structures to exert their regulatory functions, including [19,20]. Both of them have also been reported to modulate gene expression [7,21]. Based on these studies, we asked whether the G4 binding and gene expression regulation can be extended to other RBMs. RBM5 is a key member of the RBM family with reported tumor suppressive activities. Thus, we explored the potential of RBM5 in regulating the genes with G4-containing promoters.

In the current study, we identify RBM5 as a novel DNA G4-binding protein. Through correlational analyses, we discovered a strong positive correlation between RBM5 and many genes with promoter G4 structures, including the BAP1 gene. Our further analyses indicate that RBM5 binds BAP1 G4 structures and activates BAP1 gene expression. Importantly, we demonstrate that BAP1 is an essential downstream target gene of RBM5 to exert its tumor suppressive activity.

## 2. Results

### 2.1. RBM5 Is Downregulated in Breast Cancer

**Figure 1:**
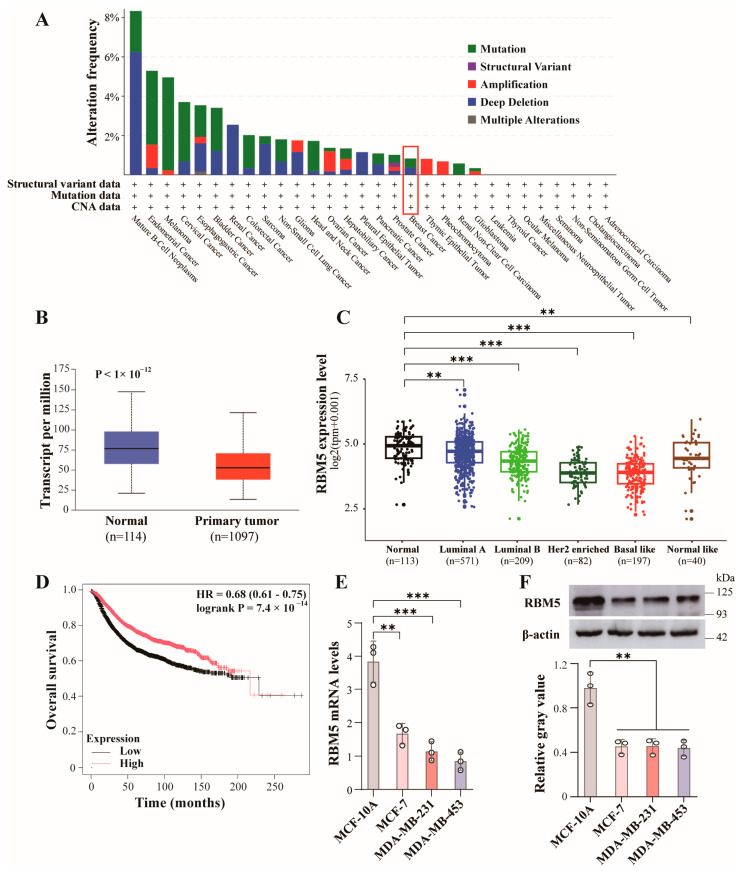
Analyses of RBM5 gene expression in breast cancer. (**A**) Genetic alterations of the human RBM5 gene in different malignancies based on the datasets of the cBioportal platform. Breast cancer is denoted by a red box. (**B**,**C**) RBM5 expression in primary breast cancer tissues and normal tissues (**B**) and in different subtypes of breast cancer samples (**C**) based on the TCGA-BRCA dataset. ** *p* < 0.01, *** *p* < 0.001. (**D**) Prognostic analysis of RBM5 expression based on an RNA-CHIP study of breast cancer patients’ samples using the Kaplan–Meier Plotter database. The probe used for the CHIP analysis is RBM5 (201394_s_at). (**E**,**F**) RBM5 expression in different mammary cell lines examined by RT-qPCR (**E**) and Western blot analysis (**F**). In (**F**), densitometric analyses of the bands after normalization by β-actin are presented under Western blot image. In (**E**,**F**) (bottom panel), data are shown as the mean ± S.D, *n* = 3 technical replicates per group across three independent biological replicates. Shapiro–Wilk test verified normal data distribution. Two-tailed unpaired Student’s *t*-test (two groups) or two-way ANOVA with post hoc multiple comparisons were used for statistical analysis. In (**F**) (top panel), representative images from three independent experiments are presented. ** *p* < 0.01, *** *p* < 0.001.

RBM5 changes have been reported in various cancers. We analyzed and compared the frequencies of RBM5 genetic alterations in 30 clinical cancers using the cBioPortal platform, which included mutations, structural variants, amplifications, deep deletions and multiple alterations. Mature B-cell tumors exhibited the highest frequency of RBM5 genetic alterations, exceeding 8% of tested tumors, while breast cancer only showed <1% of changes with mutations and deep deletions (Figure 1A). Consistently, a study based on paired non-tumor and tumor samples from 73 breast cancer patients primarily detected RBM5 alterations at the posttranscriptional level in tumor samples, including altered splicing variant levels, aberrant translational control, and reduced ubiquitination [22]. Thus, genetic alterations are not the major contributors to reduced activity of RBM5 in breast cancer.

We analyzed the TCGA-BRCA dataset consisting of 114 normal and 1097 breast cancer samples, and discovered significantly downregulated RBM5 expression in tumor samples versus normal tissues (Figure 1B). In the TCGA-BRCA dataset, compared to normal tissues, RBM5 showed reduced expression in all breast cancer subtypes, especially in the basal-like, an aggressive molecular subtype frequently overlapping with TNBC (Figure 1C). Importantly, Kaplan–Meier Plotter survival analysis revealed that elevated RBM5 expression was significantly associated with prolonged overall survival in breast cancer patients (*p* = 7.4 × 10^−14^, hazard ratio (HR) < 1; Figure 1D), indicating that RBM5 expression serves as a favorable prognostic biomarker for breast cancer patients. We also tested RBM5 expression in several mammary cell lines. Compared to the nontumorigenic human mammary MCF-10A cells, RBM5 showed reduced mRNA and protein levels in breast cancer MCF-7, MDA-MB-231 and MDA-MB-453 cells (Figure 1E,F).

### 2.2. RBM5 Negatively Regulates the Malignancy of Breast Cancer Cells

**Figure 2:**
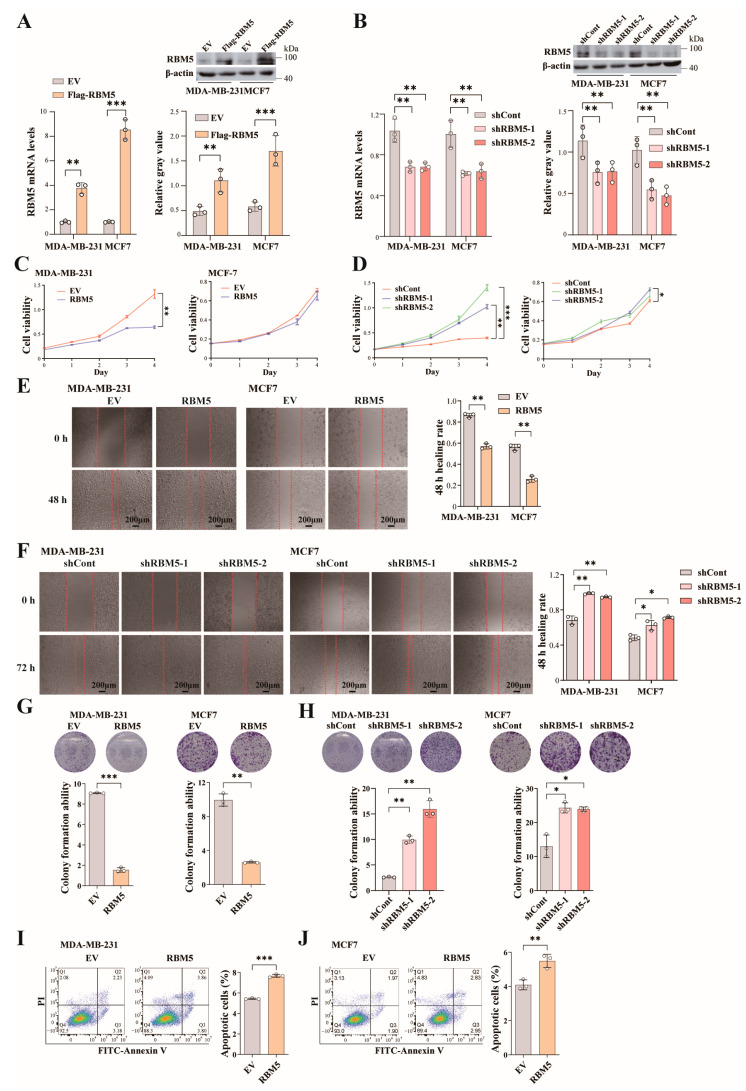
Effects of manipulated RBM5 expression on breast cancer cells. (**A**,**B**) Evaluation of ectopic RBM5 expression (**A**) and endogenous RBM5 knockdown (**B**) in MDA-MB-231 and MCF-7 cells by RT-qPCR (top panels) and Western blot analyses using RBM5 and β-actin antibodies (bottom panels). Densitometric analyses of the bands after normalization by β-actin to quantify protein expression levels are presented under Western blot images. (**C**,**D**) Effects of ectopic RBM5 expression (**C**) and endogenous RBM5 knockdown (**D**) on the viability of MDA-MB-231 and MCF-7 cells. (**E**,**F**) Scratch wound healing assay to examine the effects of ectopic RBM5 expression (**E**) and endogenous RBM5 knockdown (**F**) on the migration of MDA-MB-231 and MCF-7 cells. The quantification of cell migration is shown at the right of the images. The red dotted lines indicate the initial scratch boundary at 0 or 48 or 72-h time-points. (**G**,**H**) Clonogenic assay to test the effects of ectopic RBM5 expression (**G**) and endogenous RBM5 knockdown (**H**) on the colony formation of MDA-MB-231 and MCF-7 cells. The quantification of cell migration is shown beneath the images. (**I**,**J**) Flow cytometric analyses of the effects of ectopic RBM5 expression on the apoptotic rates of MDA-MB-231 (**I**) and MCF-7 (**J**) cells. The cells were stained with FITC-Annexin V and propidium iodide (PI) to detect early and late apoptotic cells, respectively. The quantification of cell apoptosis is shown at the right of the images. In this figure, data are shown as the mean ± S.D, *n* = 3 technical replicates per group across three independent biological replicates. Shapiro–Wilk test verified normal data distribution. Two-tailed unpaired Student’s *t*-test (two groups) or two-way ANOVA with post hoc multiple comparisons were used for statistical analysis. * *p* < 0.05; ** *p* < 0.01; *** *p* < 0.001.

To examine how manipulated RBM5 expression affected breast cancer cell proliferation, we constructed a lentiviral vector, pSL4-Flag-RBM5, and its empty vector (EV), and produced the lentiviruses. Meanwhile, we generated lentiviruses carrying two shRNAs against RBM5 (shRBM5-1 and shRBM5-2) targeting different sites of the RBM5 mRNA, and a control shRNA (shCont) with a scrambled targeting sequence. The overexpression and knockdown of these lentiviruses were validated in MDA-MB-231 and MCF-7 cells (Figure 2A,B). When tested in these breast cancer cells, ectopically expressed Flag-RBM5 could significantly reduce MDA-MB-231 cell viability compared to the EV groups, but the effects in MCF-7 cells were marginal (Figure 2C). In the experiments using shRNAs, the knockdown of endogenous RBM5 in MDA-MB-231 could significantly improve cell viability, while the effects were also significant in MCF-7 cells, but to a much lesser extent (Figure 2D). In scratch/wound healing assays, we also observed that Flag-RBM5 ectopic expression could significantly inhibit the migration of both MDA-MB-231 and MCF-7 cells (Figure 2E). Consistently, knockdown of endogenous RBM5 could enhance the migration of these breast cancer cells (Figure 2F). Additionally, in the colony formation assay, Flag-RBM5 could significantly reduce the colony numbers of MDA-MB-231 and MCF-7 cells (Figure 2G), while silencing RBM5 by the two shRNAs could markedly increase the colonies of these breast cancer cells (Figure 2H). Next, we used FITC-Annexin V and propidium iodide (PI) to stain early and late apoptotic cells, respectively, in MDA-MB-231 cells. When overexpressing Flag-RBM5, we detected elevated cell apoptosis in MDA-MB-231 cells (Figure 2I) and MCF-7 cells (Figure 2J). Together, our data of manipulated RBM5 expression indicate that RBM5 plays an antiproliferative role in breast cancer cells.

### 2.3. RBM5 Is Associated with the Expression of Genes with G4-Containing Promoters

**Figure 3:**
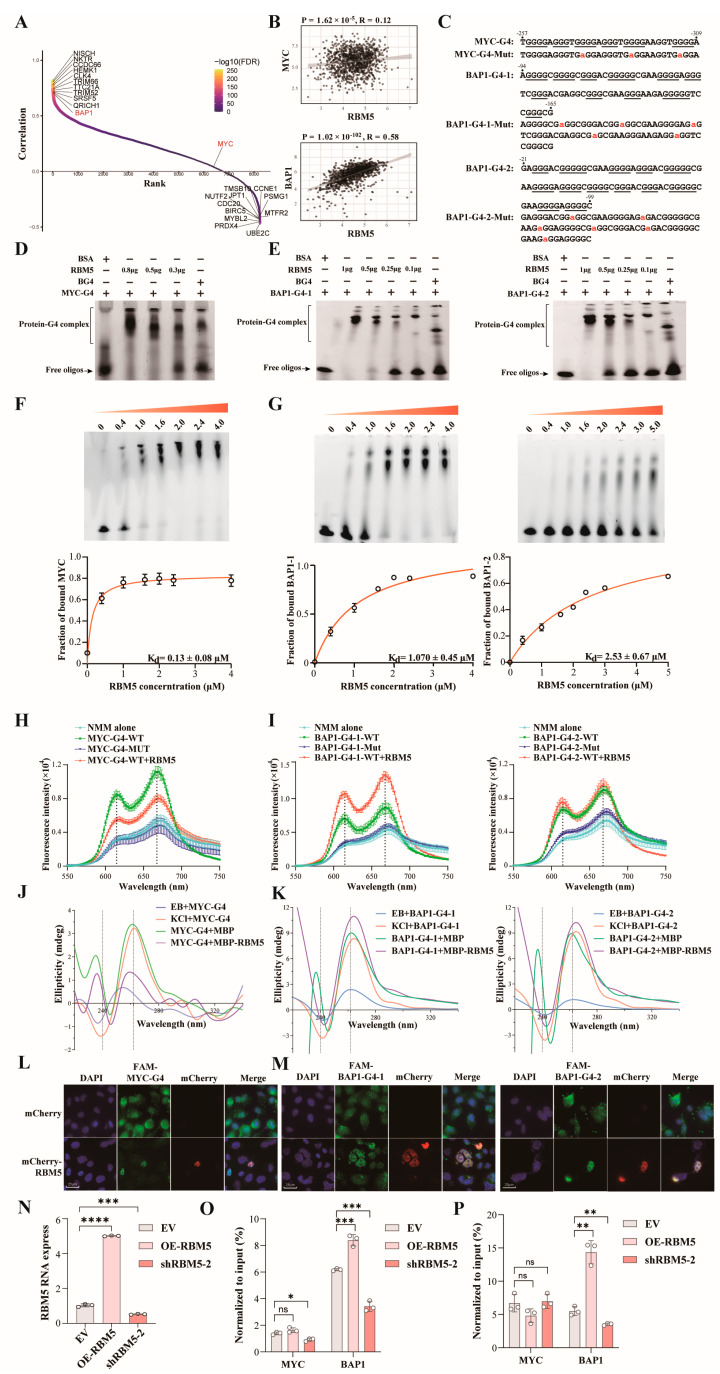
Analyses of RBM5-associated genes with G4-containing promoters. (**A**) The scatter plot of the expression correlation between the RBM5 gene and the genes with G4 motifs in the promoters. Each point represents a gene. The analyzed data are based on the TCGA-BRCA dataset. (**B**) Scatter plots of the gene expression correlation of RBM5 versus MYC (top panel) and RBM5 versus BAP1 (bottom panel). (**C**) The sequences of synthesized oligos for the MYC-G4, BAP1-G4 and their mutants. The G-tracts are underlined, and the red and lowercase letters are the mutated nucleotides. (**D**,**E**) EMSA experiments to determine RBM5 binding to MYC-G4 (**D**) and BAP1-G4 (**E**). Different amounts of RBM5, 0.5 µg of BG4 or BSA were incubated with 8 pmol of FAM-labeled oligos. The samples were resolved by native PAGE. The positions of protein-G4 complex and free oligos are denoted. (**F**,**G**) EMSA was performed to determine the binding affinity of RBM5 toward MYC-G4, BAP1-G4-1 and BAP1-G4-2 oligos. The dissociation constant (*K*_d_) values fitted from binding curves demonstrate distinct binding affinities of RBM5 for three G4 sequences. (**H**,**I**) NMM assay to examine the effects of RBM5 binding on G4 structures. An amount of 80 µg of purified His×6-RBM5 was mixed with 8 pmol of annealed WT or mutant oligos, followed by the addition of 100 pmol of NMM. The samples were scanned between 550 and 750 nm to detect the fluorescence. (**J**,**K**) Circular dichroism spectral analysis to examine the effects of RBM5 binding on G4 structures. An amount of 300 µg of purified MBP-RBM5 or MBP was mixed with 30 pmol of annealed WT or mutant oligos. The samples were scanned between 190 and 360 nm to detect the ellipticity. (**L**,**M**) Immunofluorescent staining to detect the colocalization between RBM5 and G4 structures. FAM-labeled MYC-G4 (**L**) or BAP1-G4 (**M**) oligos were co-transfected with mCherry-RBM5 or mCherry expression plasmid into MCF-7 cells. After 48 h, the cells were visualized under fluorescence microscope to capture the images. The scale bars are 25 µm. (**N**–**P**) ChIP-qPCR to determine the binding of RBM5 and associated G4 structure formation in the MYC and BAP1 promoters. (**N**) RT-qPCR to examine the overexpression (OE) and shRNA-mediated RBM5 knockdown (shRBM5-2) in MDA-MB-231 cells. ChIP-qPCR using RBM5 antibody (**O**) and BG4 antibody (**P**) to detect RBM5 enrichment and G4 structures in the MYC and BAP1 promoters. Data were subtracted from the IgG data and normalized to the input. In (**F**–**K**), data are shown as the mean ± S.D, *n* = 3 technical replicates per group. In (**O**,**P**), data are shown as the mean ± S.D, *n* = 3 technical replicates per group across three independent biological replicates. Shapiro–Wilk test verified normal data distribution. Two-tailed unpaired Student’s *t*-test (two groups) or two-way ANOVA with post hoc multiple comparisons were used for statistical analysis. * *p* < 0.05 ** *p* < 0.01; *** *p* < 0.001; **** *p* < 0.0001; ns: no significance.

RBM5 belongs to the RBM family and the proteins in this family contain the RRMs, which have been reported to bind G4 structures [23]. In this protein family, RBM4 has been reported to bind G4 structure in the promoter of the MYC gene and regulate its expression [7]. To explore whether RBM5 also possessed the activities of binding G4 and regulating gene expression, we conducted correlative gene expression analysis between RBM5 and a set of genes containing G4s in their promoters. For this purpose, the Quadron algorithm, available on GitHub (https://github.com/aleksahak/Quadron, commit: 19047e3, accessed on 5 March 2023) was used to process the genomic data in the TCGA database. Totally 8240 genes containing G4 motifs within the 1 kb sequence upstream of the transcription start site in their promoter regions were discovered to create a gene set. Pearson’s correlation analysis was performed between the genes with G4-containing promoters from this gene set and RBM5 mRNA levels, and a scatter plot was created (Figure 3A). Among these genes with G4-containing promoters showing highly positive correlation with RBM5 expression (top-left corner, Figure 3A), the NISCH, NKTR, and BAP1 genes have been reported to have tumor suppressive activities [18,24,25], while the genes highly negatively correlated with RBM5 (lower-right) contain UBE2C, PRDX4 and BIRC5 (Survivin) with oncogenic activities [26,27,28]. Notably, the MYC gene, which also contains G4 structures in its promoter, did not show a strong correlation with RBM5 (Figure 3A).

BAP1 has been identified as a deubiquitinase and acts as a tumor suppressor in various cancers [18]. In our previous study, we reported that BAP1 promoter G4 structures could activate its gene expression [13]. Meanwhile, the MYC promoter contains a G4 motif that has been well characterized for its role in regulating gene expression [9] and frequently used as a positive G4 control in many studies. When analyzing the TCGA-BRCA dataset, we found that RBM5 exhibited a significantly positive correlation with BAP1 in breast cancer samples (*R* = 0.58, *p* = 1.02 × 10^−102^). The correlation coefficient between RBM5 and MYC is *R* = 0.12 with *p* = 1.62 × 10^−5^. Despite a statistically significant *p*-value, the low correlation coefficient reflects a biologically weak association, a common phenomenon caused by large sample sizes (Figure 3B).

When analyzing the DNA sequences, we identified one G4 motif in the MYC promoter (i.e., MYC-G4) and two G4 motifs in the BAP1 promoter (i.e., BAP1-G4-1 and BAP1-G4-2) (Figure 3C). Based on their sequences, we synthesized the oligos for the MYC-G4-WT, BAP1-G4-1-WT and BAP1-G4-2-WT, as well as their corresponding mutants MYC-G4-Mut, BAP1-G4-1-Mut and BAP1-G4-2-Mut (Figure 3C and Table S1). To test the binding of RBM5 to these G4 motifs, we individually annealed MYC-G4-WT, BAP1-G4-1-WT and BAP1-G4-2-WT in the presence of 150 mM KCl, and incubated them with purified recombinant His×6-RBM5 (Figure S1A). When tested in EMSA experiments, RBM5 showed significant binding to all three tested G4 sequences in a dose-dependent manner (Figure 3D,E). Meanwhile, BG4, a G4-specific antibody, but not BSA, could also associate with these annealed G4 motifs. We next performed EMSAs using different amounts of RBM5 to determine its binding affinity to G4 sequences in the MYC and BAP1 promoters (Figure 3F,G, top panels). Nonlinear regression fitting revealed distinct dissociation constant (*K*_d_) values for the three MYC-G4, BAP1-G4-1 and BAP1-G4-2 oligos as *K*_d_ = 0.13 ± 0.08 μM, 1.07 ± 0.45 μM, and 2.53 ± 0.67 μM (Figure 3F,G, bottom panel). These quantitative data demonstrated that RBM5 possesses differential binding affinity for different promoter G4 structures.

Next, we carried out the N-methyl mesoporphyrin IX (NMM) assay to examine the G4 structures. NMM is a fluorescent molecule that selectively binds both DNA and RNA G4 structures but shows relatively weak binding to single- or double-stranded nucleic acids [29]. The annealed G4-WT and mutant oligos in Figure 3C were individually incubated with NMM, and the fluorescence of the samples was recorded using the wavelengths from 550 to 750 nm. Only the WT oligos showed emission peaks at 612 and 666 nm, with the fluorescence curves similar to that of NMM association with telomeric DNA [29]. In contrast, the three mutant oligos exhibited much reduced fluorescence that was comparable to that of NMM alone (Figure 3H,I), indicating that MYC-G4-WT, BAP1-G4-1-WT, BAP1-G4-2-WT, but not their mutant oligos, could form G4 structures. Next, we examined how RBM5 could affect the fluorescence of NMM-associated G4s. With the addition of RBM5, the MYC-G4 emission peaks at 612 and 666 nm largely decreased, indicating reduced G4 structures (Figure 3H). However, the RBM5 presence could significantly increase BAP1-G4-1-WT fluorescence at these two peaks (Figure 3I, left panel), indicating enhanced G4 structure. For BAP1-G4-2-WT, RBM5 showed marginal effects on its NMM-associated fluorescence intensity (Figure 3I, right panel). Next, the effects of RBM5 on G4 structures were also evaluated by circular dichroism spectral analysis. MYC-G4, BAP1-G4-1-WT and BAP1-G4-2-WT annealed in the presence of 150 mM KCl exhibited positive peaks at 262 nm and a negative peak at 242 nm, characteristics of G4 structures, while in the absence of KCl, the two peaks showed markedly reduced intensity (Figure 3J,K). To test the effects of RBM5 binding on these G4 oligos, a relatively high concentration of RBM5 was needed. However, purified His×6-RBM5 could only stay in relatively low concentrations, which were only enough for EMSAs, but its high concentrations would elicit protein aggregation and precipitation. To circumvent this dilemma, we fused RBM5 with maltose-binding protein (MBP) to produce recombinant MBP-RBM5 in bacteria. When purified MBP-RBM5 (Figure S1B) was mixed with the promoter G4 oligos, MYC-G4 showed largely reduced molar ellipticity at 262 nm (Figure 3J), while BAP1-G4-1-WT and BAP1-G4-2-WT exhibited increased intensity around this wavelength (Figure 3K). As a negative control, MBP alone showed marginal effects on the ellipticity at 262 nm of these G4 oligos. Together, the results of these G4-binding experiments indicate that RBM5 is capable of binding G4 structures in the MYC and BAP1 promoters but exhibits distinct effects on different G4 motifs, suggesting that RBM5 may differentially regulate the expression of different genes.

Next, we tested whether RBM5 could bind these promoter G4 oligos in cells. When mCherry-RBM5 and FAM-labeled G4 oligos were introduced into MCF-7 cells, we could observe generally colocalized fluorescent signals of MYC and BAP1 promoter G4 oligos with mCherry-RBM5 (Figure 3L,M), suggesting their association in a cellular environment.

To examine RBM5 binding to the G4 motifs of the endogenous MYC and BAP1 promoters, we carried out chromatin immunoprecipitation followed by quantitative PCR (ChIP-qPCR). MDA-MB-231 cells were infected with a lentivirus carrying an EV, expressing RBM5 or its shRNA, followed by RT-qPCR to verify RBM5 overexpression and knockdown (Figure 3N). In the ChIP assays, the RBM5 and BG4 antibodies were used with normal IgG as a control. In the qPCR, the data were subtracted from the IgG data and normalized against the input. In the ChIP-qPCR using the BAP1 antibody, RBM5 overexpression significantly increased its binding to the G4 motif region in the BAP1 promoter, but did not alter its association with the G4 region in the MYC promoter (Figure 3O). With RBM5 knockdown, RBM5 binding to the BAP1 promoter was markedly reduced, while its binding to the MYC promoter also showed a significant reduction, but to a much lesser extent (Figure 3P).

Collectively, these ChIP-qPCR results demonstrate that RBM5 binds to the endogenous G4 motifs in the BAP1 promoter and subsequently facilitates the G4 structure formation. Meanwhile, RBM5 can physically interact with the G4 structures in the MYC promoter; its binding does not significantly alter the MYC-G4 structure.

### 2.4. RRM Domains of RBM5 Are Involved in G4 Binding

**Figure 4:**
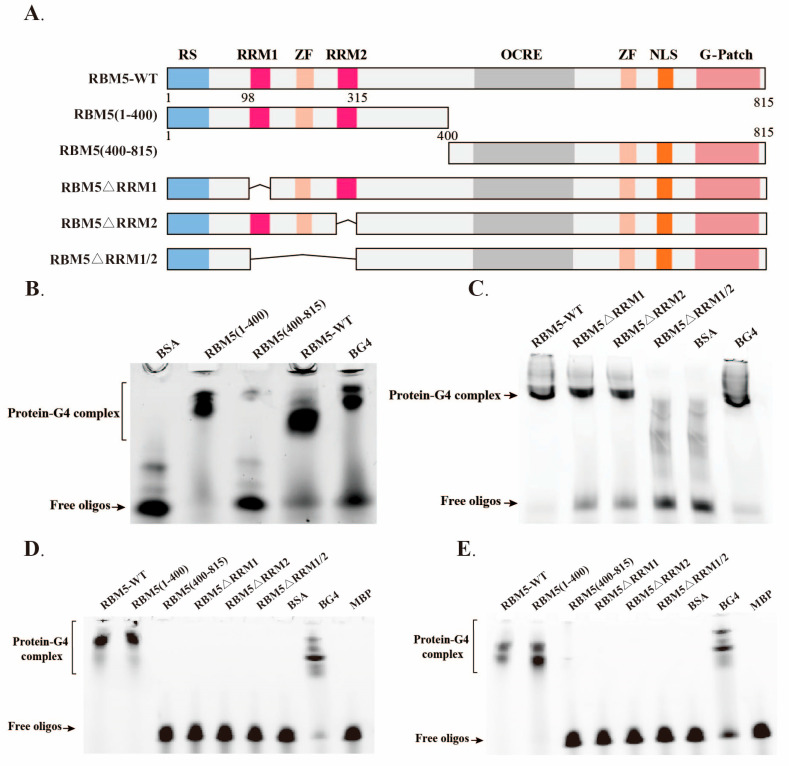
Determination of the binding domains of RBM5 to G4 structures. (**A**) Domain structure diagrams of RBM5 WT and mutant proteins. The functional domains are indicated at the top. RS: arginine/serine-rich, RRM: RNA recognizing motif, ZF: zinc finger, OCRE: OCtamer Repeat, NLS: nuclear localization signal. (**B**–**E**) EMSA analyses to determine the association of His×6-RBM5 WT and its mutants with FAM-labeled MYC-G4 (**B**,**C**) and BAP1-G4 (**D**,**E**) oligos. The positions of protein-G4 complex and free oligos are indicated.

To determine the regions of RBM5 responsible for G4 binding, we generated several RBM5 mutants (Figure 4A). Among them, RBM5 (1–400) encloses both RRM1 and RRM2, while RBM5 (400–815) is the C-terminal region. Another three mutants are the RBM5ΔRRM1, RBM5ΔRRM2 and RBM5ΔRRM1/2 with deleted RRM1, RRM2, or both RRMs, respectively. When examining the binding of RBM5 WT and mutants with MYC-G4 using EMSAs, we found that RBM5-WT and RBM5 (1–400), but not RBM5 (400–815), could show slowly migrated bands (Figure 4B). Additionally, both RBM5ΔRRM1 and RBM5ΔRRM2 mutants, but not RBM5ΔRRM1/2, showed detectable interactions with MYC-G4 (Figure 4C). As controls, the G4 antibody BG4, but not BSA, could also bind MYC-G4 in EMSAs. These results indicate that at least one of the RRM1 or RRM2 of RBM5 is required for its MYC-G4 binding activity.

Next, we tested the binding of RBM5 WT and mutants with BAP1-G4-1-WT and BAP1-G4-2-WT oligos. In the EMSA experiments, only RBM5-WT and RBM5(1-400), but not any of the other RBM5 mutants, showed bands with slow migration (Figure 4D,E). The data indicated that both RRMs are required for RBM5 to bind the two G4 motifs in the BAP1 promoter.

### 2.5. Promoter G4 Motifs Are Required for RBM5-Mediated Gene Expression

**Figure 5:**
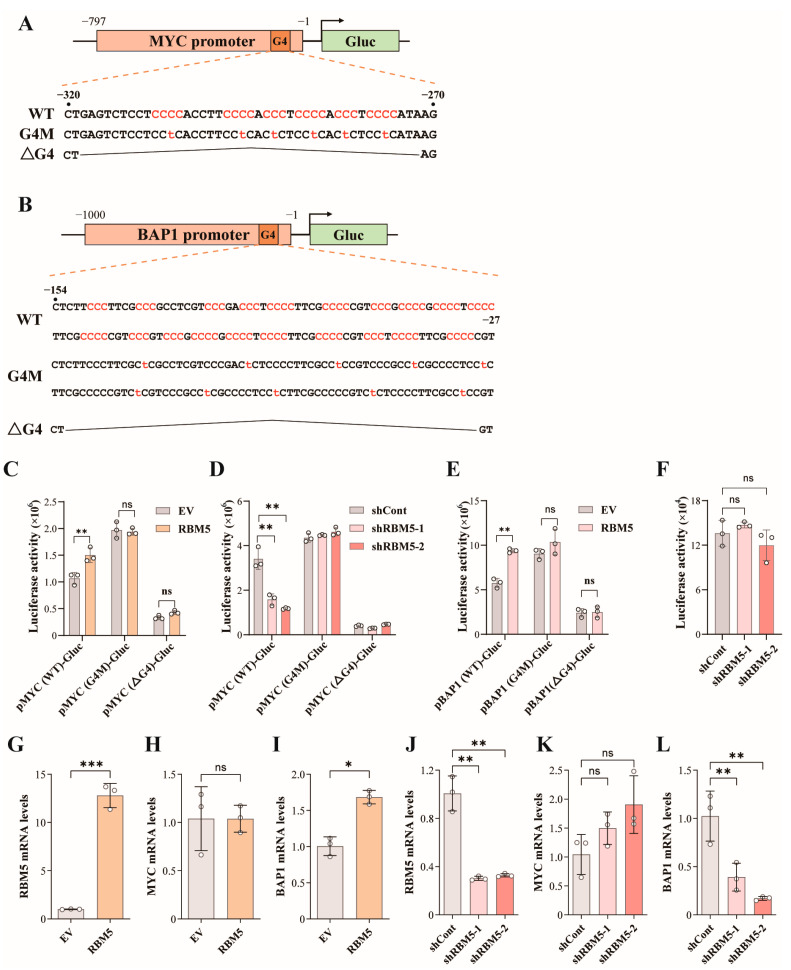
Examination of the effects of manipulated RBM5 expression on MYC and BAP1 expression. (**A**,**B**) Diagrams of MYC (**A**) and BAP1 (**B**) promoter Gluc reporters. The WT, G4M and ΔG4 sequences are presented. (**C**–**F**). Effects of ectopic RBM5 expression (**C**,**E**) and endogenous RBM5 knockdown (**D**,**F**) on the expression of Gluc reporters driven by the promoters containing MYC-G4 (**C**,**D**) and BAP1-G4 (**E**,**F**). WT, G4M or ΔG4 reporter plasmids and pCMV-SEAP were co-transfected into HeLa cells. Gluc activity was measured and normalized against SEAP activity. The data are shown as mean values ± S.D. from triplicated samples and the experiments were repeated three times with similar results. ns: no significance; ** *p* < 0.01. (**G**–**L**) Effects of manipulated RBM5 expression on endogenous MYC and BAP1 gene expression. With ectopic RBM5 expression (**G**) or RBM5 knockdown by shRBM5-1 and shRBM5-2 (**J**) in MDA-MB-231 cells, expression of endogenous MYC (**H**,**K**) and BAP1 (**I**,**L**) was evaluated by RT-qPCR. Data are shown as the mean ± S.D, *n* = 3 technical replicates per group across three independent biological replicates. Shapiro–Wilk test verified normal data distribution. Two-tailed unpaired Student’s *t*-test (two groups) or two-way ANOVA with post hoc multiple comparisons were used for statistical analysis. ns: no significance; * *p* < 0.05; ** *p* < 0.01; *** *p* < 0.001.

To evaluate how manipulated RBM5 expression could change the activities of these two G4-containing promoters, we generated reporter constructs, pMYC(WT)-Gluc and pBAP1(WT)-Gluc, that used the MYC promoter and BAP1 promoter, respectively, to drive Gluc expression. Meanwhile, we also created the mutant reporters containing the promoters with a deleted G4 motif (ΔG4) and a mutated G4 motif (G4M) (Figure 5A,B). When cotransfected HeLa cells with the reporter constructs and Flag-RBM5 or its empty vector (EV), Flag-RBM5 could significantly increase the Gluc activity of pMYC(WT)-Gluc compared to the EV (Figure 5C). However, for the pMYC(ΔG4)-Gluc and pMYC(G4M)-Gluc reporters, no significant Gluc activity change was observed between Flag-RBM5 and EV (Figure 5C), suggesting the MYC-G4 motif is responsible for RBM5 binding to drive MYC expression. Next, when the reporter was cotransfected with shRBM5-1 and shRBM5-2, pMYC(WT)-Gluc exhibited reduced Gluc activity (Figure 5C), while the two mutant reporters showed no response to RBM5 knockdown (Figure 5D). Similarly, when ectopically expressing Flag-RBM5, we observed increased pBAP1(WT)-Gluc activity versus the EV control, while pBAP1(ΔG4)-Gluc and pBAP1(G4M)-Gluc did not respond to Flag-RBM5 expression (Figure 5E). However, with the knockdown of endogenous RBM5 by shRNAs, pBAP1(WT)-Gluc did not show detectable changes in Gluc activity (Figure 5F). Notably, in these reporter assays, compared to the WT reporters of both MYC-G4 and BAP1-G4, the corresponding G4M reporters showed increased Gluc activity, while the ΔG4 reporters manifested markedly reduced activity (Figure 5C–E). We also examined how RBM5 changes affected the expression of endogenous MYC and BAP1 genes. With ectopically expressed RBM5 in MDA-MB-231 cells (Figure 5G), we did not detect changes in endogenous MYC mRNA (Figure 5H), but observed significantly increased endogenous BAP1 levels (Figure 5I). When RBM5 was knocked down by its shRNAs (Figure 5J), the endogenous MYC gene showed no significant changes (Figure 5K), but BAP1 markedly decreased (Figure 5L).

Together, the results of the reporter assays demonstrated that RBM5 enhances the activity of the G4-containing promoters of both the MYC and BAP1 genes. However, altered RBM5 only impacts the expression of the endogenous BAP1 gene, but not MYC. The promoter G4s may serve as binding motifs of RBM5 to stimulate gene expression. The mutations of G4 motifs may increase gene expression due to the removal of G4s’ steric hindrance, but complete deletion of G4-containing regions may remove essential elements in the promoters, leading to significantly reduced promoter activity.

### 2.6. BAP1 Is a Key Downstream Target of RBM5 to Suppress Breast Cancer Malignancy

**Figure 6:**
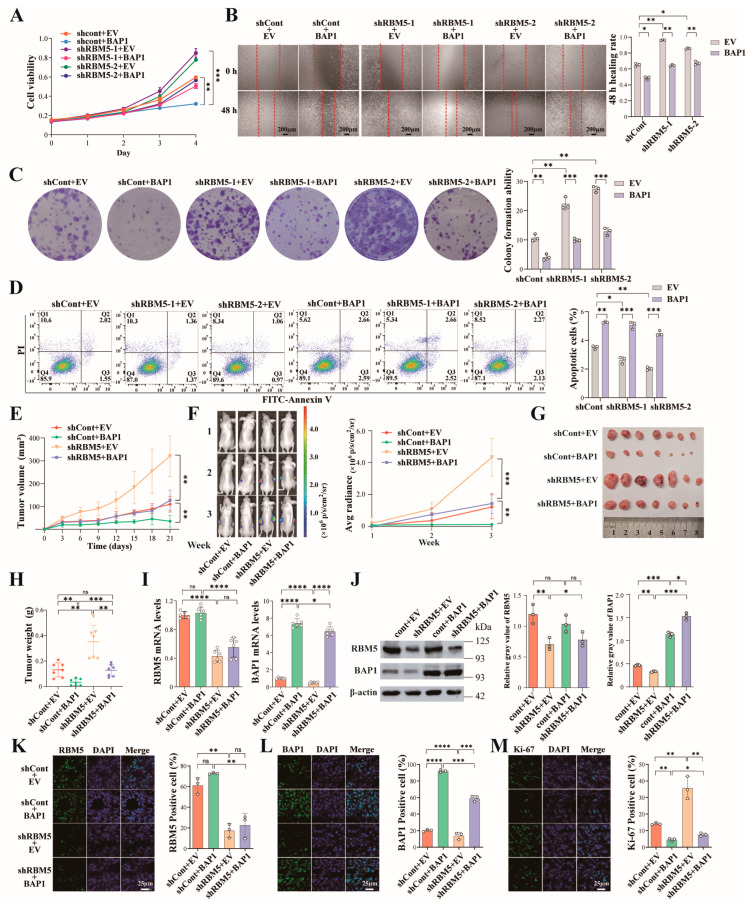
Evaluation of BAP1 contribution to RBM5 knockdown-promoted breast cancer malignancy. (**A**–**D**) Determination of the effects of RBM5 knockdown and BAP1 overexpression on the viability (**A**), migration (**B**), colony formation (**C**), and apoptotic rate (**D**) of MDA-MB-231 cells. Lentiviruses carrying shRBM5-1, shRBM5-2, shCont, pSL4-BAP1, or pSL4 EV were used to infect MDA-MB-231 cells. Cell proliferation was measured by CCK8 assay, followed by the calculation of cell viability (**A**). Other assays were carried out as described in Section 4. (**E**–**L**) Tumor xenograft study to determine the effects of RBM5 knockdown and BAP1 overexpression on breast cancer tumor formation. After MDA-MB-231 cells with RBM5 knockdown and/or BAP1 overexpression were inoculated into nude mice, the growth of xenografted tumors was monitored by measuring tumor sizes to get the tumor volumes (**E**) and tumor bioluminescence using luciferin (images at left and quantitation at right) (**F**). When the mice were sacrificed and tumors were excised, the tumor images were captured (**G**) and tumor weights (**H**) were obtained. The RBM5 knockdown and BAP1 overexpression were verified by RT-qPCR (**I**), Western blot analysis (**J**), and immunofluorescence staining (**K**,**L**). In (**J**), densitometric analyses of the bands after normalization by β-actin are presented at the right Western blot images. The expression of Ki-67 was also detected by immunofluorescence staining (**M**). In (**A**–**D**), data are shown as the mean ± S.D, *n* = 3 technical replicates per group across three independent biological replicates. In (**E**–**L**), data are presented as mean ± S.D. Each group contained five mice (*n* = 5 biological replicates), with three technical replicates for each sample. Shapiro–Wilk test verified normal data distribution. Two-tailed unpaired Student’s *t*-test (two groups) or two-way ANOVA with post hoc multiple comparisons were used for statistical analysis; ns: no significance; * *p* < 0.05, ** *p* < 0.01, *** *p* < 0.001, **** *p* < 0.0001.

As we discovered that BAP1 is one of the most positively correlated genes containing promoter G4s, we next evaluated how BAP1 contributed to RBM5-regulated breast cancer development. In MDA-MB-231 cells expressing RBM5 shRNAs and pSL4 empty vector (shRBM5 + EV), we could see significantly improved cell viability compared to the cells carrying shCont and EV (shCont + EV) (Figure 6A). When BAP1 was introduced into the cells expressing shRBM5-1 or shRBM5-2 (shRBM5 + BAP1), the cell viability was significantly reduced to levels comparable to those of shCont + EV. Additionally, the MDA-MB-231 cells of the shCont + BAP1 group exhibited significantly lower viability than the shCont + EV cells (Figure 6A). Similarly, in wound healing assays, enhanced cell migration caused by RBM5 knockdown could also be significantly attenuated by ectopic BAP1 expression (Figure 6B). In colony formation assays, BAP1 expression reduced the colony numbers that were increased by RBM5 knockdown (Figure 6C). When the cells co-stained by Annexin V and PI were analyzed by a flow cytometer, RBM5 knockdown-elicited apoptotic cell reduction could be restored by BAP1 overexpression (Figure 6D). These data indicate that BAP1 is an essential downstream target gene of RBM5 to exert its tumor suppressive activity.

To validate the in cellulo observation, we carried out in vivo studies using a mouse xenograft model. MDA-MB-231 cells expressing shRBM5-2 or shCont, and carrying the BAP1 expression cassette or EV, were inoculated into the left and right flanks of female BALB/c nude mice, followed by monitoring the tumor growth. Based on the tumor volumes, RBM5 knockdown by shRBM5-2 (shRBM5-2 + EV) could cause markedly increased tumor growth when compared to the control (shCont + EV) group (Figure 6E). With ectopic BAP1 expression (shRBM5-2 + BAP1), the enhanced tumor growth by RBM5 knockdown was significantly reduced, compared to the shRBM5-2 + EV groups. Consistently, the mice of the shCont + BAP1 group exhibited the slowest tumor growth rate among all four groups (Figure 6E). With intraperitoneally injected luciferin, we observed the same tumor growth trends among the four groups based on the relative bioluminescent intensity of the xenograft tumors (Figure 6F). After sacrificing the mice and excising the tumors, we found the tumor weights showed consistent results with the tumor growth curves (Figure 6G,H). We examined the tumors by RT-qPCR and Western blot analysis, and observed the corresponding RBM5 knockdown and BAP1 overexpression in the four samples (Figure 6I,J). When analyzing the tumors by immunofluorescence staining, we could also verify successful RBM5 knockdown in tumors expressing shRBM5-2 (Figure 6K) and increased BAP1 expression in tumors carrying pSL4-BAP1 (Figure 6L). Importantly, the mice with the shRBM5-2 + EV treatment exhibited the strongest Ki-67 signal, which could be markedly dampened by overexpressed BAP1 in the shRBM5-2 + BAP group (Figure 6M).

Together, the results of the in cellulo and in vivo functional studies demonstrated that RBM5 is a bona fide tumor suppressor in breast cancer development, and BAP1 is a primary downstream target of RBM5 in breast cancer.

## 3. Discussion

Many previous studies have demonstrated the essential roles of RBMs in cancer development by rewiring RNA metabolism at multiple levels, such as alternative RNA splicing, mRNA polyadenylation, RNA nuclear–cytoplasmic transport, RNA modifications, and RNA stability [4]. Their dysregulation, including mutations, altered expressions, and post-translational modifications, promotes various cancer hallmark traits, including sustained proliferation, immune evasion, metastasis, and therapy resistance. All these positions RBMs as both emerging biomarkers and potential therapeutic targets.

Although RBMs primarily regulate RNA-related processes, several reports have demonstrated their activities of DNA binding and regulation. RRMs are the characteristic structures of the RBM family and are responsible for associating with RNA molecules. Interestingly, RRM domains are also reported to bind DNA, including single-stranded telomeric DNA [30] and G4 DNA [23]. Niu et al. demonstrated that RBM4/LARK could bind to the G4 structures in the promoters of multiple genes and facilitate their formation and stability, leading to enhanced gene expression [7]. However, whether any other RRM protein is capable of regulating gene expression remains elusive and deserves extensive exploration.

Previous studies indicate that RBM5 regulates alternative splicing and exerts a tumor suppressive role in multiple cancers, such as lung cancer [31], prostate cancer [32], and medulloblastoma [33]. Currently, whether RBM5 regulates the development and progression of breast cancer has not been systematically investigated, especially in animal models. In this study, we first validated the tumor suppressive activity of RBM5 in breast cancer and then investigated the potential function of RBM5 in regulating gene expression. Due to the reported G4-binding affinity of RRMs, we asked whether RBM5 could regulate gene expression through binding to promoter G4 structures. When analyzing a set of genes with G4-containing promoters, we obtained multiple tumor suppressive genes that showed strong positive correlation with RBM5, while a number of oncogenes were negatively correlated with RBM5, consistent with RBM5′s tumor suppressive activity. Among the top positively correlated genes, we identified the BAP1 gene for further exploration due to its G4-regulated expression reported by us [13]. As a control, we included the G4 motif in the promoter of the MYC gene, which showed no statistical correlation with the RBM5 in gene expression. When analyzing RBM5 binding to the G4 structures, we discovered differential effects of RBM5 on MYC-G4 and BAP1-G4 using different molecular biology approaches. The two RRMs of RBM5 also showed different contributions to MYC-G4 and BAP1-G4, suggesting RBM5′s sequence specific binding activity to G4-containing promoters. In the functional studies using in cellulo approaches and a mouse model, we demonstrated that ectopic BAP1 expression could abolish the stimulative effects of RBM5 knockdown on cell proliferation, suggesting that BAP1 is a primary downstream target of RBM5 in breast cancer development.

In the reporter assay, we noticed that mutated G4 motifs could increase the reporter activity, while deletion of the G4 motifs showed a significant decrease in Gluc. These results suggested that the G4 motif regions play an important role in activating gene expression. The G4 structures in the promoters may recruit transcription factors to activate the transcription of the downstream gene bodies. As previously reported, MYC-G4 positively regulates its gene expression [9]. We also reported that MAZ binds to the G4s in the cyclin D1 promoter to activate its gene expression [15]. However, G4 presence in a promoter may not only act as a unique structure to recruit transcription factors and activate the target gene, but also may physically hamper gene transcription. As we and other groups have reported, promoter G4 motifs negatively regulate the expression of YY1, HRAS, and BCL2 [8,10,12]. Mutations that disrupt G4 motifs in the MYC and BAP1 promoters may remove G4′s steric hindrance and create a relatively relaxed structure, which favors transcription through a different and G4-independent mechanism. G4 motifs have been detected in the promoters of the genes that play key regulatory roles in cell proliferation and differentiation [34]. Both the folding/unwinding of G4 structures and their recruitment of transcription factors can dynamically determine the expression levels of these genes. Thus, G4 motif mutations or deletion created the statuses that the genes become uncontrollable by G4-binding proteins.

We observed positive responses of both the MYC promoter and BAP1 promoter to RBM5 in the reporter assays; however, altered RBM5 expression could only change the expression of the endogenous BAP1 gene, but not the MYC promoter. The data are consistent with the strong gene expression correlation of RBM5 with BAP1, but not MYC (Figure 3A,B). ChIP-qPCR results also revealed that altered RBM5 expression marginally affected BG4 enrichment on the MYC promoter (Figure 3P). However, in the EMSA experiments, RBM5 exhibited higher binding affinity to MYC-G4 than to BAP1-G4s, while the binding curve of RBM5 and MYC-G4 was very different from those of its binding to BAP1-G4s (Figure 3F,G). We predict that the following reasons may contribute to these discrepancies. First, the region close to the MYC-G4 locus could be occupied by other G4 binding proteins, such as CNBP, hnRNPA1, and nucleolin [35,36,37]. The association of these proteins in breast cancer cells may competitively prevent or reduce the accessibility of RBM5 to the MYC-G4 locus. Second, the distinct binding patterns of RBM5 to MYC-G4 and BAP1-G4s may also determine the different regulatory effects. RBM5 uses two RRMs to bind each of the two BAP1-G4s, but one of its RRMs is sufficient to bind MYC-G4 (Figure 4D,E). Third, luciferase reporter plasmids have inherent experimental limitations. Reporter plasmids exist as extrachromosomal circular DNA in transfected cells, lacking the native chromatin context, such as histone modifications and chromatin-associated regulatory proteins that the endogenous promoters have, and thus may not faithfully recapitulate the complex transcriptional microenvironment in cellulo.

Interestingly, although RBM5 can bind both MYC-G4 and BAP1-G4 oligos when tested in different assays, its effects on the G4 structures from the two promoters are distinct. Based on the NMM assay and circular dichroism analyses, RBM5 could reduce MYC-G4 formation but enhance the structures of BAP1-G4. Meanwhile, we also discovered that the presence of either RRM1 or RRM2 is sufficient for RBM5 to bind MYC-G4, but both RRMs are needed for RBM5 binding to BAP1-G4s. These data suggest that RBM5 recognizes the G4 structures from these two genes through different mechanisms. As we demonstrated above, the RBM5 gene shows a strong positive correlation with BAP1 in breast cancer samples, RBM5 positively regulates the expression of the endogenous BAP1 gene, and both RRMs are needed for RBM binding to BAP1-G4; however, none of these could be observed between RBM5 and MYC. Thus, we predicted that the simultaneous binding of the two RRMs to BAP1-G4s may lead to relatively stable RBM5-G4 association and a genuine impact on BAP1 gene expression, which cannot be achieved on the MYC promoter.

It is noteworthy that BAP1 is one of the RBM5′s top positively associated genes with G4-containing promoters. We cannot exclude that other members in this gene set showing either strong positive or strong negative correlation with RBM5 may also be activated or repressed by RBM5, respectively. Whether RBM5 regulates the expression of additional genes through binding to their promoter G4 structures deserves future investigation.

Overall, in the current study, we determined RBM5 as a bona fide tumor suppressor in breast cancer, verified RBM5 as a promoter G4 binding protein, and discovered BAP1 as a novel target gene of RBM5. Importantly, we also observed differential G4 binding effects and transcriptional regulation of RBM5 in the promoters of the BAP1 and MYC genes. Our findings extend the understanding of RBM5-regulated cellular activities from RNA metabolic processes to gene transcription.

## 4. Materials and Methods

### 4.1. Electrophoretic Mobility Shift Assay

Electrophoretic mobility shift assays (EMSAs) were performed to verify the binding between target proteins and double-stranded DNA (dsDNA) or G4 structures. For the protein-G4 binding assay, FAM-labeled oligonucleotide (oligo) containing G4 motifs was first annealed at a concentration of 4 μM in an annealing buffer consisting of 50 mM Tris-HCl, 50 mM KCl and 40% (*w*/*v*) PEG 200. All binding reactions were performed on ice for at least 1 h. Native polyacrylamide gel electrophoresis (PAGE) was conducted in an ice bath using a constant voltage of 100 V. For protein-dsDNA binding, the samples were separated on 8% native PAGE for 50 min, while for protein-G4 binding, the samples were resolved on 8% native PAGE containing 100 mM KCl and 40% (*w*/*v*) PEG 200 for 10–14 h. After electrophoresis, the gels were scanned and imaged using a Typhoon FLA7000 imaging system (GE Healthcare, Boston, MA, USA). The sequences of all probes or oligos used in EMSA experiments are listed in Table S1.

### 4.2. Luciferase Reporter Assays

To determine the effects of G4s on promoter activity, reporter constructs were generated containing WT, G4 motif-deleted, or G4 motif-mutated promoters to drive the expression of Gaussia luciferase (Gluc). In reporter assays, HeLa cells cultured in 24-well plates were cotransfected with 100 ng of the reporter constructs. When testing the effects of RBM5 knockdown or overexpression on the reporters, 100 ng of pSL4-Flag-RBM5 or shRBM5, respectively, were cotransfected with each reporter with empty vector (EV) or shCont as controls. pCMV-SEAP (20 ng) expressing secreted alkaline phosphatase (SEAP) was also cotransfected as an internal control. Forty-eight hours post-transfection, aliquots of medium from the transfected wells were collected to measure Gluc activity, then normalized against the SEAP activity in the same sample. In each well, the Gluc activity was normalized against the reading of SEAP.

### 4.3. Cell Culture, Transfection, Lentiviral Production and Infection

HeLa and HEK-293T cells were cultured in DMEM (Gibco, Thermo Fisher Scientific, Waltham, MA, USA, Cat# 11965092) supplemented with 10% fetal bovine serum (FBS, ExCellBio, Shanghai, China, Cat# FSP500). MDA-MB-231 cells were cultured in RPMI 1640 (Gibco, Waltham, MA, USA, Cat# 11875093) containing 10% FBS. MCF-7 cells were cultivated in MEM (Gibco, Waltham, MA, USA, Cat# A1451801) containing 10% FBS. All cell lines were incubated at 37 °C in a humidified incubator with 5% CO_2_. The authenticity of all cell lines was verified via the short tandem repeat (STR) profiling assay. For transient transfection, cells were transfected with the indicated plasmids using Lipofectamine 2000 (Thermo Fisher Scientific, Waltham, MA, USA, Cat# 11668019) following the manufacturer’s instructions. The procedures for lentiviral production, lentiviral infection of target cells, and subsequent antibiotic selection were performed following the procedure described in our previous publication [38].

### 4.4. Plasmid Construction

shRNAs were designed as described in our previously reported strategy [39]. The target sequences used for knockdown of human RBM5 are GGTGATTCAAGGAAA-GCACATT and GGAGGCAGTGTTGACTACAGTT. A scrambled sequence (GGGAC-TACTCTATTACGTCATT) was used to generate a control shRNA (shCont). All shRNAs were subcloned into a lentiviral vector that could be used for both transient transfection and lentiviral infection.

To create overexpression vectors, total RNA was extracted from MCF-10A cells using the TRIzol reagent (Thermo Fisher Scientific, Waltham, MA, USA, Cat# 15596026) and cDNA was synthesized by reverse transcription based on the total RNA using random hexamers as primers. The coding sequences (CDs) of the RBM5 and BAP1 genes were amplified by PCR using the cDNA as template, specific primers (Table S1), and Phanta EVO HS super-fidelity DNA polymerase (Vazyme, Nanjing, China). The amplified fragments were then inserted into the expression pSL4 lentiviral vector driven by the CMV promoter. Meanwhile, the promoter regions of MYC (−797 to −1, with the transcription start site designated as +1 and the first nucleotide of its upstream as −1) and BAP1 (−1000 to −1) from the MCF-10A cell genomic DNA were also amplified by PCR. The amplified promoters were inserted upstream of the Gaussia luciferase CDS in a basic reporter vector pGluc.

### 4.5. Reverse Transcription and Quantitative PCR (RT-qPCR)

Reverse transcription and quantitative PCR (RT-qPCR) were performed to quantify the relative expression levels of target genes. For reverse transcription, 2 μg of total RNA was reverse-transcribed into complementary DNA (cDNA) using oligo(dT) primers and M-MLV reverse transcriptase (Vazyme, Nanjing, China, Cat. No. R021-01). Quantitative PCR was conducted using LightCycler 480 SYBR Green PCR Master Mix (Roche, Basel, Switzerland, Cat. No. S4438) and gene-specific primers (Table S1) on a LightCycler 480 instrument (Roche, Basel, Switzerland). The relative expression level of each target gene was normalized against the internal reference gene β-actin, and the data were calculated using the 2^−ΔΔCT^ method.

### 4.6. Protein Expression and Purification

All recombinant proteins were expressed in *E. coli* BL21 (DE3). The bacterial cultures were grown at 37 °C with constant shaking until the optical density at 600 nm (OD_600_) reached 0.6, followed by induction of protein expression by 0.15 mM IPTG at 18 °C overnight. The harvested bacteria were resuspended in ice-cold lysis buffer (20 mM HEPES, 0.2 mM EDTA, 100 mM KCl, 20% glycerol, 1% Triton X-100, 2 mM PMSF, 1 mg/mL lysozyme, pH 8.0). After ultrasonic disruption on ice, the bacterial lysate was subjected to centrifugation at 12,000× *g* for 30 min at 4 °C to collect the soluble fraction. Recombinant proteins with a His×6 tag were affinity-purified using Ni-NTA agarose beads (GE Healthcare, Chicago, IL, USA) and the bound proteins were eluted with lysis buffer supplemented with 400 mM imidazole. Maltose-binding protein (MBP)-tagged recombinant proteins were purified using Amylose Resin (New England Biolabs, Ipswich, MA, USA) and eluted with elution buffer containing 10 mM maltose. The molecular weight and purity of all purified recombinant proteins were verified by SDS-PAGE.

### 4.7. Lentivirus Production and Infection

For lentivirus production, 293T cells were used following our previously published procedure [40]. For lentiviral infection, breast cancer cells were individually plated at a confluency of about 40% in cell culture dishes with complete medium and cultured overnight. Lentivirus was added to the culture medium along with 8 μg/mL polybrene (Sigma-Aldrich, St. Louis, MO, USA, Cat# H9268), and left in the cell culture incubator for 6 h [40]. After infection, the cells were selected using 1 μg/mL puromycin in the culture medium.

### 4.8. Circular Dichroism Study

The circular dichroism assays were performed on a spectropolarimeter (Chirascan, Applied Photophysics Ltd., Surrey, UK). Oligonucleotides (oligos) were diluted to 4 μM in 50 mM Tris-HCl buffer with 150 mM KCl or without it. The samples were annealed at 95 °C for 5 min and gradually cooled down to 25 °C at a rate of 0.01 °C/s. For the protein-G4 binding assay, annealed oligos were incubated with about 300 μg of purified protein. All binding reactions were incubated on ice for at least 1 h, and then scanned in a quartz cuvette with 0.5 cm optical path length to acquire the CD spectra from 190 to 360 nm with a rate of 1 nm/s at 25 °C.

### 4.9. N-Methyl Mesoporphyrin IX Analysis of G4 Structure

The experiments were carried out following the protocol in our recent publication [41]. N-methyl mesoporphyrin IX (NMM) is a molecule specifically binding and stabilizing G4 structures. With the conformational changes caused by NMM binding, G4 structures can be excited at the wavelength of 399 nm, and are excited with strong fluorescence at the characteristic emission wavelengths of about 612 nm and 666 nm. In the current study, 80 nM of DNA oligos annealed in the presence of 150 mM KCl were added into the wells of a black flat-bottom 96-well plate. Purified proteins were added to the wells to a final concentration of 6 μM, followed by the addition of NMM solution to achieve a final concentration of 1 μM, with a total reaction volume of 100 μL per well. After incubation at ambient temperature for 1 h in the dark, the samples were excited at 399 nm, and fluorescence spectra were recorded within a wavelength range of 550 to 750 nm.

### 4.10. Immunofluorescence Staining

Cells were seeded on coverslips placed in 12-well cell culture plates and cultured overnight. The following immunostaining treatments of the cells were carried out at ambient temperature unless specified. The cultured cells were fixed with the Immunol Staining Fix Solution (Beyotime, Shanghai, China, Cat# P0098) for 30 min, followed by blocking with 10% FBS for 30 min. The cells were then incubated with a primary antibody for 30 min. After washing three times with PBS, the cells were incubated with Alexa Fluor 488-conjugated secondary antibodies for 1 h. Next, the cells were washed three times with PBS again, counterstained by DAPI to visualize cell nuclei, and imaged using a DMi8 Thunder Imager Live Cell System (Leica Microsystems, Wetzlar, Germany). For IF staining of frozen tissue sections, 10 μm thick frozen sections were prepared and fixed with 4% paraformaldehyde (PFA) for 20 minutes. The fixed sections were blocked with 10% goat serum at 37 °C for 1 h and then incubated with the primary antibody overnight at 4 °C. Subsequently, the sections were washed three times with PBS and incubated with Alexa Fluor 488-conjugated secondary antibodies at 37 °C for 1 h. After washing three times with PBS, the sections were counterstained with DAPI, and images were acquired using the DMi8 Thunder Imager Live Cell System (Leica Microsystems, Wetzlar, Germany).

### 4.11. Chromatin Immunoprecipitation-Quantitative PCR (ChIP-PCR)

Ten million cells were crosslinked with 1% formaldehyde at room temperature for 10 min, and the reaction was quenched by adding 125 mM glycine. The crosslinked cells were collected and lysed in nuclear lysis buffer containing 50 mM Tris-HCl, pH 8.1, 10 mM EDTA, 1% SDS, and 1× protease inhibitors to isolate nuclei. The nuclei were then subjected to ultrasonic treatment to shear chromatin DNA into fragments with an average length of 300–500 bps. The DNA fragments were incubated with 2 μg of the BG4 antibody recognizing G4 structures (Absolute Antibody, Oxfordshire, UK, Cat# Ab00174-1.1), or BAP1 antibody (Abcepta, San Diego, CA, USA, Cat# AD80566) with normal IgG as a control, followed by precipitation with Protein A/G magnetic beads (Thermo Fisher Scientific, Waltham, MA, USA, Cat# 88803) at 4 °C overnight. Next, the magnetic beads were sequentially washed with four different buffers to remove non-specifically bound impurities: Buffer I (0.1% SDS, 1% Triton X-100, 2 mM EDTA, 20 mM Tris-HCl, pH 8.1, 150 mM NaCl, 1× protease inhibitors), Buffer II (identical to Buffer I except using 500 mM NaCl), Buffer III (0.25 M LiCl, 1% NP-40, 1% sodium deoxycholate, 1 mM EDTA, 10 mM Tris-HCl, pH 8.1), and a TE buffer (10 mM Tris-HCl, pH 8.0, 2 mM EDTA, 1× protease inhibitors). After the washing, the beads were resuspended in a buffer containing 50 mM NaHCO_3_, 1% SDS, and crosslinks were reversed by incubation at 65 °C for 5 h. The released DNA fragments were treated with RNase A and proteinase K to remove contaminating RNA and proteins, respectively. The purified DNA was extracted using phenol–chloroform and precipitated with ethanol. The immunoprecipitated DNA from all samples and 10% of the input was analyzed by qPCR using the primer pairs that cover the G4 motifs in the MYC or BAP1 promoter (Table S1). The data from the ChIP samples by BG4 and BAP1 antibodies were subtracted from the corresponding IgG data and then normalized by the input.

### 4.12. Wound Healing Assay

MCF-7 and MDA-MB-231 cells with RBM5 knockdown or overexpression were seeded in 6-well plates and cultured for 24 h until reaching 80% monolayer confluency. A scratch wound was made by scraping the cell monolayer with a plastic pipette tip, followed by continuous culture in fresh medium. Microscopic images were captured at 0 h (immediately after wounding), 48 h and 72 h post-wounding. Cell migratory capacity was qualitatively evaluated based on the residual wound area at the end of the experiment.

### 4.13. CCK8 Cell Proliferation Assay

MDA-MB-231 and MCF-7 cells with different treatments were seeded in 96-well plates at a density of 1500 cells per well. Subsequently, 10 μL of CCK-8 solution was added to each well, and the plates were incubated at 37 °C for 3 h. The absorbance at 490 nm was measured sequentially over 4 days.

### 4.14. Cell Colony Formation

Cells were infected with lentivirus, and the infected cells were seeded in 12-well plates at a density of 1000 cells per well. After about 10–14 days, the cells were fixed with 10% formalin and stained with 0.5% crystal violet. The plates were then imaged, and the number of colonies in each well was counted.

### 4.15. Flow Cytometry Analysis

Cells were infected with lentivirus, and subsequent apoptosis assays were performed using the FITC-Annexin V Apoptosis Detection Kit (Vazyme, Nanjing, China, Cat# A211-02) following the manufacturer’s instructions. Apoptotic cells were analyzed by flow cytometry (Beckman, A00-1-1102, Brea, CA, USA). FITC-Annexin V and propidium iodide (PI) included in the kit were used to detect early and late apoptotic cells, respectively.

### 4.16. Mouse Xenograft Study

Animal experimental protocols were approved by the Animal Care and Ethics Committee of Northeast Forestry University. MDA-MB-231 cells (5 × 10^6^) stably expressing firefly luciferase (Fluc) were infected by lentiviruses carrying an empty vector, or expressing BAP1, shRBM5, or shRBM5/BAP1. The transfected cells were resuspended in 100 μL of serum-free RPMI 1640 medium mixed with Matrigel (BD Biosciences, Bedford, MA, USA) at a 1:1 volume ratio. A total volume of 200 μL of the cell suspension was subcutaneously inoculated into the left and right flanks of BALB/c nude mice. Tumor sizes were measured every 3 days using a vernier caliper (Mitutoyo Corporation, Kawasaki, Japan), and tumor volume (V) was calculated with the formula: V = length × (width^2^)/2. To image the tumors weekly, mice were anesthetized prior to intraperitoneal injection of luciferin substrate at a dose of 150 mg/kg body weight. Ten min post injection, the mice were placed in an in vivo imaging system (PerkinElmer, Waltham, MA, USA) for bioluminescent imaging. Three weeks post-cell inoculation, the mice were humanely euthanized. All tumor xenografts were harvested, photographed, and subsequently subjected to immunofluorescence staining, Western blot, and RT-qPCR analyses for further detection.

Five-week-old female BALB/c nude mice were purchased from Beijing Weitong Lihua Experimental Animal Technology Co., Ltd. (Beijing, China). Based on preliminary and present tumor growth data, Cohen’s d effect sizes of key pairwise comparisons were calculated: shRBM5+EV versus shCont+EV (d = 3.37), shCont+BAP1 versus shCont+EV (d = 3.96), and shRBM5+BAP1 versus shRBM5+EV (d = 2.83). A priori power analysis via G*Power 3.1 (α = 0.05, power = 0.80) revealed that the minimal sample sizes required for the above comparisons were 3, 3 and 4 mice per group, respectively. Following the reduction principle of animal ethics to cut down the number of animals under sufficient statistical power, we finally allocated 5 of these 5-week-old female BALB/c nude mice to each experimental group.

### 4.17. Statistical Analysis

All experimental data were obtained from at least three independent biological experiments with consistent results, with three replicates per group, and all results are presented as the mean ± standard deviation (SD). The Shapiro–Wilk normality test confirmed that all data conformed to a normal distribution. A two-tailed unpaired Student’s *t*-test was used for pairwise comparisons between two groups. For multifactorial analyses, two-way analysis of variance (ANOVA) was performed, followed by post hoc multiple comparison tests to evaluate intergroup differences. Statistical significance was set at *p* < 0.05. The levels of significance were denoted as * *p* < 0.05, ** *p* < 0.01, *** *p* < 0.001, and **** *p* < 0.0001, respectively. *p* > 0.05 was considered no significant difference (n.s.).

Correlation analyses between RBM5 expression and the expression levels of 8240 G4-motif-containing genes were performed using the R 4.2. For each gene, Spearman’s rank correlation coefficient (ρ) and its uncorrected *p*-value were calculated using the cor.test function with the argument exact = FALSE to handle ties in TPM values. The 95% confidence intervals (CIs) for Spearman’s ρ were computed based on Fisher’s Z-transformation. To control the false discovery rate (FDR) arising from multiple parallel tests across the 8240 genes, uncorrected *p*-values were adjusted using the Benjamini–Hochberg (BH) procedure via the *p*.adjust function. A two-sided adjusted *p*-value (FDR) < 0.05 was considered statistically significant.

## Data Availability

The raw data supporting the conclusions of this study are available from the corresponding author upon request. The bioinformatics data used in this study were obtained from the TCGA database through the following public platforms: http://ualcan.path.uab.edu/ (accessed on 15 May 2025) and https://kmplot.com/analysis/ (accessed on 2 November 2025).

## Supplementary Materials

The following supporting information can be downloaded at: https://www.mdpi.com/article/10.3390/molecules31142492/s1, Figure S1: SDS-PAGE analyses of purified recombinant proteins. Table S1: Sequences of oligonucleotides used in PCR and construction of shRNAs.
