# Supplementary material for: RBM5 Acts as a Tumor Suppressor in Breast Cancer Through Binding to G-quadruplexes in the BAP1 Gene Promoter to Activate Its Expression
Source: Molecules. 2026 Jul 16;31(14):2492. doi: 10.3390/molecules31142492 (PMC13415644; doi:10.3390/molecules31142492)
Supplement: Supplementary file 1 [file molecules-31-02492-s001.zip › Supplementary Figure S1.pdf]

Supplementary Figure S1

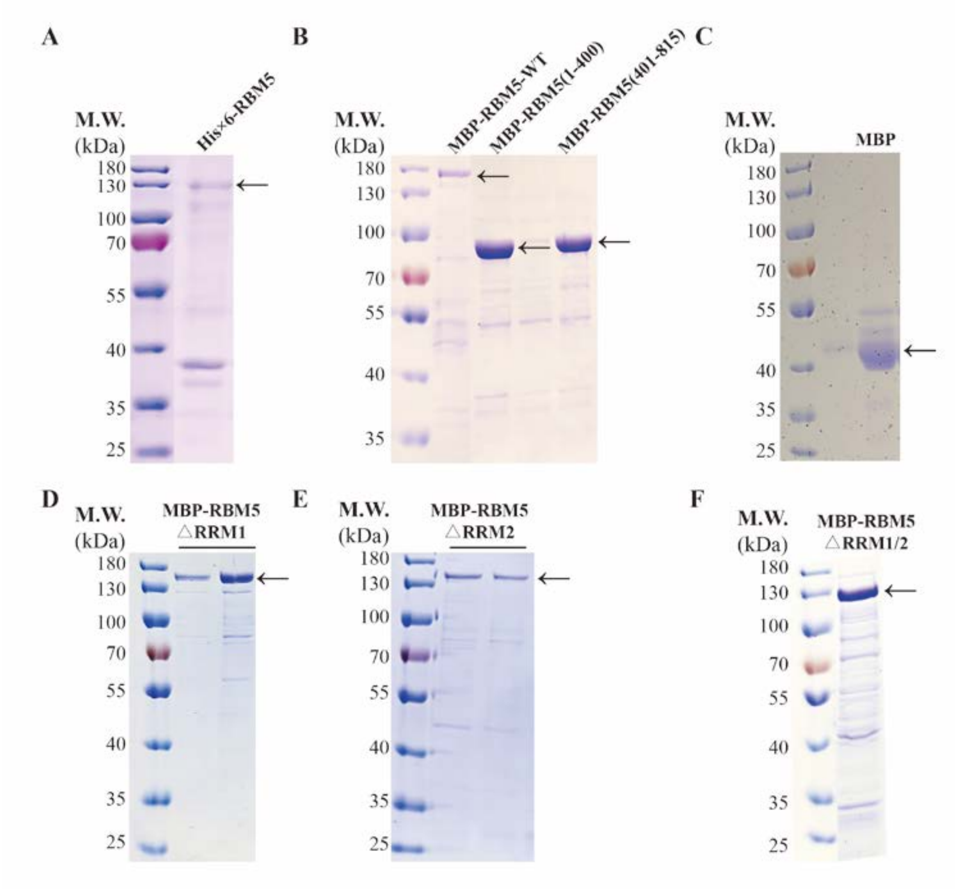

Supplementary Figure S1. SDS-PAGE analyses of purified recombinant proteins.

Purified His×6-RBM5 (A), MBP-RBM5-WT and its mutants MBP-RBM5-(1-400), MBP-RBM5-(401-815) (B), MBP (C), MBP-RBM5-(ΔRRM1) (D), MBP-RBM5-(ΔRRM2) (E), and MBP-RBM5-(ΔRRM1/2) (F) were analyzed.
