# Supplementary material for: RBM5 Acts as a Tumor Suppressor in Breast Cancer Through Binding to G-quadruplexes in the BAP1 Gene Promoter to Activate Its Expression
Source: Molecules. 2026 Jul 16;31(14):2492. doi: 10.3390/molecules31142492 (PMC13415644; doi:10.3390/molecules31142492)
Supplement: Supplementary file 1 [file molecules-31-02492-s001.zip › Supplementary Table S1.pdf]

Supplementary table S1. Sequences of oligonucleotides used in PCR and construction of shRNAs

No.	Name	Sequence (5’ – 3’)	Application
1	MYC-G4-WT	TGGGGAGGGTGGGGAGGGTGGGGAAGGTGGGGA	
2	BAP1-G4-1-WT	AGGGGCGGGGCGGGACGGGGGCGAAGGGGAGGGTCGGGACG<br>AGGCGGGCGAAGGGAAGAGGGGGTCCGGGCG	
3	BAP1-G4-2-WT	GAGGGACGGGGGCGAAGGGGAGGGACGGGGGCGAAGGGGAG<br>GGGCGGGGCGGGACGGGACGGGGGCGAAGGGGAGGGGC	
4	MYC-G4-Mut	TGGGGAGGGTGaGGAGGGTGaGGAAGGTGaGGA	EMSA, NMM, circular dichroism studies
5	BAP1-G4-1-Mut	AGGGGCGaGGCGGGACGGaGGCGAAGGGGAGaGTCGGGACGA<br>GGCGaGCGAAGGGAAGAGGaGGTCCGGGCG	
6	BAP1-G4-2-Mut	GAGGGACGGaGGCGAAGGGGAGaGACGGGGGCGAAGaGGAGG<br>GGCGaGGCGGGACGaGACGGGGGCGAAGaGGAGGGGC	
7	BAP1-939U17	AGAGGAGGCGGCTGGTT	
8	BAP1-1172L18	GGTGGGCGGACTGGAACT	
9	MYC-803U24	AGGAGGAACAAGAAGATGAGGAAG	
10	MYC-900L24	TCCAGCAGAAGGTGATCCAGACTC	
11	β-actin-876U20	TTCCTTCCTGGGCATGGAGT	RT-qPCR quantitation
12	β-actin-1063L20	TCTTCATTGTGCTGGGTGCC	
13	RBM5-2085U20	GCTGGAAGCCTTGGAGCTAA	
14	RBM5-2199L20	AAACTGCTTCTTGCGCTTGG	
15	RBM5-492-H3-L	CCACAAGCTTGAATATGTGCTTTCCTTGAATCACCAAACAAGG<br>CTTTTCTCCAAGGGATA	
16	RBM5-492-2a	AGCTTAATGTGCTTTCCTTGAATCACCTTTTTG	shRBM5-1
17	RBM5-492-2b	AATTCAAAAAGGTGATTCAAGGAAAGCACATTA	
18	RBM5-1057-H3-L	CCACAAGCTTGAATACTGTAGTCAACACTGCCTCCAAACAAGG<br>CTTTTCTCCAAGGGATA	
19	RBM5-1057-2a	AGCTTAACTGTAGTCAACACTGCCTCCTTTTTG	shRBM5-2
20	RBM5-1057-2b	AATTCAAAAAGGAGGCAGTGTTGACTACAGTTA	
21	U6-BamHI-U	CATCGGGATCCGACGCCGCCATCTCTAGG	Universal primer for shRNA construction
22	MYC-pt-(-198)U20	GTGGGCGGAGATTAGCGAGA	
23	MYC-pt-(-344)L20	AGAGCTAGAGTGCTCGGCTG	ChIP qPCR

No.	Name	Sequence (5' – 3')	Application
24	BAP1-pt-(-181)U18	CCTCAGCGCTCAGCATCG	ChIP qPCR
25	BAP1-9L18	CATGCGCTCGAAGGCGAA	
